# Does clinical method mask significant VTE-related mortality and morbidity in malignant disease?

**DOI:** 10.1038/sj.bjc.6605091

**Published:** 2009-06-02

**Authors:** A Maraveyas, M Johnson

**Affiliations:** 1 Queen's Centre for Oncology and Hematology, Hull and York Medical School, Department of Academic Oncology, Castle Hill Hospital, Castle Road, Hull HU16 5JQ, UK; 2 Hull and York Medical School and St Catherine's Hospice, Throxenby Lane, Scarborough, North Yorkshire YO12 5RE, UK

**Keywords:** vascular thromboembolism, mortality

## Abstract

After more than 150 years of a recognised link between cancer and vascular thromboembolic events (VTE), and despite a greatly improved understanding of its pathophysiology, epidemiology and treatment, the management of patients with cancer and VTE is still limited. Limitations can be related to the thromboembolism itself, the underlying cancer, or to the management process. There is significant literature that deals with the first two, but very little regarding the systems we use, or how the inadequacies in documentation, identification and classification of VTE affect the cancer patients themselves. This review aims to raise awareness of this neglected area and stimulate research that may lead to improvements in patient care.

The relationship between thrombosis and cancer was studied by Trousseau in the nineteenth century; however, limitations in clinical management persist despite subsequent therapeutic and diagnostic strides.

The first limitation observed is that patients still die from clinically apparent vascular thromboembolic event (VTE), even if diagnosed and treated appropriately, irrespective of whether it is cancer related or not.

The second limitation lies with the complex management challenges that result from the cancer itself, increasing the risk of a poorer VTE outcome even if diagnosed and treated appropriately. The underlying cancer may also influence the perception of the treating physician, affecting decision making for a patient with cancer-related VTE. This issue is exacerbated by the tendency to practise suboptimal management despite best evidence.

Finally, the third limitation arises from the clinical features of VTE that can render it ‘invisible’ using current processes, which include ‘index of clinical suspicion’ and diagnostic and reporting tools. This could lead to a ‘perception gap’, which compounds the first two limitations.

This review provides a summary of existing evidence, discusses gaps in our understanding and suggests avenues of research. We present the topic under two themes: (A) Visible VTE and (B) Invisible VTE.

## (A) Visible VTE

*Limitations because of thrombosis* Vascular thromboembolic events carry a risk of death, whether they are cancer-related or not (Summary Box 1). Regression models have been developed for pulmonary embolism (PE), myocardial infarction (MI) and cerebrovascular accident (CVA) in the general acute medical population from which mortality-prediction tools are derived. These tools highlight that visible thrombosis (i.e., diagnosed), despite presumably optimal treatment, can cause significant mortality.

In addition, the manifestation of one-type VTE is a harbinger of further vascular thromboembolic events, for example, during the first year after a PE there is a threefold increased risk of CVA and a 2.5-fold risk of MI (Sørensen *et al*, 2007). Thrombosis-affected cancer patients treated with anticancer therapies in an adjuvant setting will survive long enough to experience this ‘domino’ effect.

*Limitations because of cancer*

Cancer related: Vascular thromboembolic event in patients with cancer can be difficult to manage due to an increased risk of bleeding and further episodes of VTE despite anticoagulation, particularly with warfarin (Prandoni *et al*, 2002). These risks increase with progressive cancer (Prandoni *et al*, 2002), and clinical decisions can be difficult in patients for whom the focus of treatment is palliative, but who are not imminently dying (Johnson and Sherry, 1997; Noble, 2007). The risks worsen with advanced disease not only because of ulceration or venous compression by tumour but also because of significant disseminated intravascular coagulation (Johnson *et al*, 1999b). Patients may also have thrombocytopenia secondary to treatment or marrow infiltration.

Therapy related: Anticancer therapies increase the risk of VTE. However, apart from low level evidence base recommendations for myeloma patients receiving thalidomide and linalinomide, there is no clear evidence-based guidance regarding thromboprophylaxis.

Clinician related: There is an ambivalent attitude to VTE in cancer setting, which may lead to underdiagnosis and undertreatment. It may be commented, ‘a big PE is a nice way to go’ (Noble *et al*, 2008a), although evidence does not support this assumption (Havig, 1977). The unpredictable nature of recurrent VTE may also be a problem for patients for whom ‘last goals’ are important, and a sudden but unpleasant death can contribute to family distress in bereavement. As clinical decision making moves from traditional paternalism towards patient centre, we need to consider both RCT evidence base for management and patient view of what they consider acceptable. Significant differences may exist between the health professional and the patient with regard to risks and benefits.

It is recognised that suboptimal management occurs despite best evidence. Knowledge of research evidence is rarely enough to change practice (NHS Centre for Reviews and Dissemination, 1999) and other factors that affect clinical decision making. Level 1 evidence-based guidelines for secondary prevention of VTE in cancer patients, including those with advanced disease, recommending long-term treatment with low-molecular-weight heparin (LMWH), have been published (Lyman *et al*, 2007; Noble *et al*, 2008b). Despite this, a UK VTE Registry's most recent report noted that only 9% of patients received LMWH for more than 30 days and only 5% for more than 90 days (VERITY Venous Thromboembolism Registry, 2007). The use of warfarin persists in cancer patients despite the risks of polypharmacy, liver metastases, low albumin, inadequate nutritional status and chemotherapy scheduling.

*Limitations because of current recognition processes* The ambivalent attitude regarding the significance of cancer-related VTE may affect the ‘index of suspicion’ and willingness to investigate a possible VTE, even when clinically apparent. It may also be reflected in the recording process in clinical trials, which in turn is reflected in subsequent evidence-based clinical guidance, leading to a vicious cycle of poor recognition and suboptimal management.

Limitations due to recording of VTE in clinical trials: Highly sensitive, accurate and minimally invasive imaging techniques such as spiral computed tomography should improve the detection of VTE. VERITY figures reflect this, but research studies, at least until recently, do not. In a systematic review of 19 major advanced pancreatic cancer (APC) randomised trials published from 1997 to 2007, including 6212 patients (Sgouros and Maraveyas, 2008), VTE incidence was inconsistently reported.

Possibly, trial eligibility criteria select patients in whom the VTE incidence is truly low. Thrombosis may occur in the diagnosis phase of pancreatic cancer, rendering the patient ineligible for treatment trials. Patients with cardiovascular events during the preceding 6 months may also be excluded. However, it remains surprising that this selected population, with a tumour known to be highly thrombogenic, apparently has a negligible reported VTE incidence during a median survivorship of 6 months. The more likely explanation is that VTE is not seen as a drug-related adverse event. Therefore, the data may be recorded, but not reported in the publication. The primary published results of the NCI-CTG PA.3 trial (Moore *et al*, 2007a) did not mention VTE incidence, but on request, figures of 14% all-type VTE were reported (Moore *et al*, 2007b). Vascular thromboembolic events formally reported as the cause of death in only two of 1447 patients (0.1%) (Sgouros and Maraveyas, 2008). Even if a significant number of VTE have silent or ‘sudden death’ presentations, such a low incidence of reported VTE-related death is surprising.

**Figure 1:**
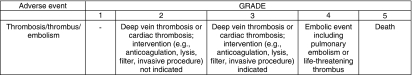
Current CTCAE grading system used for thromboembolism.

Limitations due to the method of recording VTE in clinical trials: Binary reporting, i.e. VTE present or absent (Moore *et al*, 2007b), is better than none at all, but does not capture clinical complexities. A grading system documenting type, complexity and outcome of VTE is desirable. We suggest that the currently used CTCAE tool (Common Terminology Criteria for adverse events version 3.0; the National Cancer Institute (NCI) Cancer Therapy Evaluation Programme (CTEP)) for VTE (Figure 1) attempts to grade, but is wholly inadequate and has no evidence base. For example, a 50-year-old patient with an asymptomatic, small PE found on interval imaging has a different outcome risk compared with the 70-year-old patient with a saddle embolus and circulatory–neurological compromise, but both would be CTCAE grade 4.

There are a number of scoring systems for PE outcome in the general medical literature. One prognostic model (Aujesky *et al*, 2006) classifies PE patients into five risk classes (I–V), with different cumulative 90-day mortalities, from 0% in class I to 24.4% in class V.

Some of the immediate mortality from a diagnosed VTE may be captured as a CTCAE grade 5 event within ongoing serious adverse event (SAE) reporting, but much mortality or morbidity can be misattributed and subsequent deaths or morbidity are unlikely to be recorded as VTE related. A grading system that captures clinical consequence is urgently needed.

Limitations in attributing causality to cancer treatments: Although almost all systemic cancer treatments can increase the risk of VTE, the commonly reported causality of VTE as an SAE in trials remains cancer. Therefore, the incidence of VTE for some new agents, for example, erythropoietin (Bennett *et al*, 2008), was underestimated, only becoming apparent through pharmacovigilance review. As the pro-thrombotic tendency of thalidomide had already been exposed, (Bennett *et al*, 2006) this was immediately recognised with linalinomide and included in the licensed product pamphlet. However, a prolonged post-market pressure was exerted before this was included in the thalidomide drug information too.

It is a recent appreciation, rather than during drug development, that conventional chemotherapy agents increase VTE risk by 4–6 fold (Ogren *et al*, 2006). We therefore have little comparative VTE risk data for commonly used agents or for the effect of different ways of administration on this risk. The need for prospective data can be shown; ECF chemotherapy for gastoesophageal cancer produced an incidence of 17.7% VTE in an RCT setting (Starling *et al*, 2007), whereas a retrospective database analysis of earlier RCTs using the same regimen from the same group presented a year earlier recorded only 3%.

Newer anti-angiogenic agents are being subjected to greater scrutiny, possibly because VTE prominence was recognised during developmental work and also, perhaps, because of potentially large liability damages. Trials with bevacizumab, for example, have recorded VTE incidence more carefully (Kindler *et al*, 2005).

## Consequences of visible VTE

Malignancy-related VTE reduces survival (Alcalay *et al*, 2006; Mandalà *et al*, 2007). Most studies are retrospective registry related, but the striking effect of VTE on APC can be seen most clearly in the sub-analysis of the NCI-CTG PA3 trial (Moore *et al*, 2007b). The detrimental effect of VTE on survival is greater than the beneficial impact of erlotinib and has the same impact as performance status and stage. Vascular thromboembolic event is more common in advanced stages of cancer but confers a worse prognosis even if corrected for pathological stage. Stage-related distinctions in APC are difficult, but in colorectal cancer (CRC), in which the majority of patients have accurate pathological staging, there is increased VTE-related mortality irrespective of stage (Alcalay *et al*, 2006). The greatest impact of VTE was seen in Dukes A (TNM stage I) patients despite potentially less arduous surgery and no chemotherapy. It is suggested that the underlying malignancy has a more aggressive course, despite no difference in conventional prognostic indicators such as histological grade. However, the separation of the curves within 100 days after VTE is in contrast to the expected natural history of resected Dukes A cancer. We think this raises significant doubt that the VTE-related poorer prognosis is simply because VTE is the preserve of advanced disease or is an epiphenomenon of more aggressive disease. Vascular thromboembolic event can have lethal consequences and, although rarer in early-stage disease, the impact is greater in such patients. This chimes with the sub-analyses of existing trials (Akl *et al*, 2007) of LMWH in cancer, suggesting that patients with better prognoses received greater survival benefit from LMWH. In advanced disease, it may be more difficult to discern the contribution of VTE to mortality over and above that of cancer. Stage IV CRC patients from the above study had no statistically significant different survival, despite a trend for worse outcome (Alcalay *et al*, 2006).

## (B) Invisible VTE

*Epidemiological and pathological evidence* The true prevalence of VTE is underestimated because many are clinically unapparent (Summary Box 2). Pre-mortem studies indicate that when a proximal DVT is diagnosed, PE has occurred in up to 50% of patients, although only 33–40% are symptomatic (Khorana and Fine, 2004; Ogren *et al*, 2006; Cronin *et al*, 2007). Postmortem and epidemiological studies in cancer patients have established VTE both as a common coexisting entity and as a direct cause of death. An autopsy study investigating the presence of VTE in patients with various types of cancer, including 441 patients with APC, found that 42% of the APC patients had PE, the sole cause of death in 14% of patients (Ogren *et al*, 2006). This study verifies other data identifying APC patients to be at high risk of VTE (Maraveyas *et al*, 2007). The development of biological markers predictive of VTE risk would be a major step forward. Some recent work in early stages suggests that cancer tissue levels of tissue factor (TF) or circulating TF antigen may hold such promise (Khorana *et al*, 2007).

The risk of VTE increases with advanced tumour. A study of consecutive admissions to a palliative care unit showed evidence of lower limb DVT in 135 out of 258 patients (52%; 95% confidence interval 46–58%) (Johnson *et al*, 1999a). Only 22 patients (9%) had clinically recognised VTE and confirmation with imaging.

Second, there is evidence that VTE mimics other conditions and thus is unrecognised rather than truly asymptomatic. Postmortem studies confirm a persistent poor pre-mortem recognition of PE (Cronin *et al*, 2007), ranging from 11% in the 1950s to 45% in the 1990s. Although reliable twenty-first century data are unavailable, it is likely that more than half the patients, including those with cancer, dying from PE today are undiagnosed pre-mortem.

*Incidentally discovered VTE* The high-resolution CT scanner has produced the phenomenon of incidental VTE, found in up to 1.5% of routine helical CT scans, increasing to 2.6–3.4% in patients with cancer and as high as 6.3% for all-type VTE (Cronin *et al*, 2007). Some of these may remain silent if left untreated; however, some will become symptomatic with either an atypical presentation or misattributed to the underlying morbidity. There may be an opportunity to initiate effective treatment, but some may result in an unexpected mortality through persistent misrecognition or from sudden death. Incidental PE is not necessarily clinically insignificant and a lethal PE is not always preceded by a ‘silent’ phase; Havig's postmortem study showed that two-thirds of the abruptly dead had had symptoms of ‘advertising emboli’ (Havig, 1977).

Splanchnic thrombosis is another poorly understood entity and rarely clinically suspected. Most studies are dominated by retrospective surgical series, primarily in benign disease. Thus, we know little about the incidence, natural history, prognosis or management of these events in cancer patients. It may be more common than previously thought, with more incidental splanchnic DVTs found than ileofemoral or common iliac DVTs during staging CT (Sgouros and Maraveyas, 2008) in patients with APC. Thus, the natural history of splanchnic DVT, its potential role as a marker of more serious problems such as ascites, variceal haemorrhage, bowel insufficiency and necrosis, needs further elucidation.

*Is incidental silent?* The difference between the terms ‘incidental’ and ‘silent’ is pertinent. A VTE may be discovered incidentally, but may not be silent, as confirmation may lead to discovery of symptoms. A silent VTE is one that has no symptoms or signs that could reasonably be attributable to VTE even in retrospect.

A retrospective case–control study of (O'Connell *et al*, 2006) 59 patients with incidental PE showed that patients with PE were significantly more likely to be fatigued (odds ratio of 4.82 (*P*=0.0002)). Breathlessness, a classic symptom of PE, was seen in a minority of cases (22 *vs* 8%, *P*=0.02). However, as this study included cancer patients at different stages of diagnosis and treatment, it is difficult to discern which symptoms are due to cancer or to its treatment, especially when the symptoms may be atypical of PE and the time of embolism is unknown.

## Consequences of invisible VTE

*Hidden mortality* We have coined the term ‘early death burden’ (EDB) to describe an observed early (12 week) survivorship shortfall in patients enrolled in randomised phase III trials (Sgouros and Maraveyas, 2008). Early death burden seems most conspicuous in advanced APC; 23% compared with under 4% in metastatic CRC. The conventional explanation is that this is due to the ‘aggressive’ nature of APC (short survival time and reduced chemosensitivity of APC compared with CRC). However, given the eligibility criteria designed to enter patients with an expected survival of 3 months or more by specifying adequate end-organ function and good performance status, it is no more likely that almost a quarter of participating APC patients will die of disease progression within 12 weeks than those with CRC. Early death burden may be favourably affected by systemic treatment efficacy or conversely exacerbated by some of the anticancer treatments themselves. Some of it will be caused by ‘early toxic death’ of the treatment and some will reflect cancer-related deaths from infection or organ failures. However, despite these factors, we speculate that there is still a shortfall in expected survival in APC, which we suggest reflects the existence of significant unappreciated VTE. Vascular thromboembolic event-driven EDB is likely to be a mixture of invisible events, that is, sudden death with no postmortem or those who die with misattributed symptoms, and events from ‘visible’ VTE, the cause of death being mislabeled as ‘cancer progression’. Such hidden events may be prevented by LMWH (in a secondary prevention dose), and recruitment into a phase IIb trial of thromboprevention in APC with EDB as a secondary endpoint is now closed (EUDRACT No.:111-111111-11). Data from this study will help confirm or refute our concerns.

## Conclusion

The presence of cancer and its treatment make diagnosis and management of VTE difficult. There are gaps in our understanding regarding the clinical impact of VTE in a cancer patient and the interplay between cancer, its treatment and thrombosis, which could lead to ambiguity and complacency over its recognition, diagnosis, treatment and recordable outcome. We suggest that this could contribute to preventable morbidity and mortality of cancer patients, especially in those with highly thrombogenic tumours. We hope to cast some doubt on the explanation that the survivorship shortfall in patients with VTE is solely an epiphenomenon of a more aggressive malignancy or chemo-resistant tumours. We have made some suggestions that may help direct further efforts to inform this field (Box 3).

A clinically relevant grading system for recording VTE in cancer trials, systematic study of the clinical impact of co-incidental/silent VTE and continued research into optimal anticoagulation are urgently needed to close some of these gaps. A higher level of clinical suspicion, a greater understanding of the complexities of cancer-related VTE, with strategies for prevention, recognition and management, and research into developing biological predictive markers of VTE are required to improve the current situation. If not, Trousseau's legacy will continue, exposing the shortcomings of the contemporary clinical method.

## **Box 1** Visible VTE challenges

• Limitations because of the thrombosis

 ○ Natural history of VTE includes mortality even in non-cancer patients despite current best treatment

• Limitations because of the cancer

 ○ The cancer patient (e.g., hemorrhage from primary, effect of secondaries e.g., liver, marrow, performance status compromise)

 ○ Clinician's perception of benefit of treatment

 ○ Delay in implementing evidence-based standards

• Limitations because of clinical processes and clinical trial method

 ○ Low-clinical index of suspicion and clinician reluctance to investigate

 ○ Binary recording is insufficient and current CTCAE grading tool not fit for purpose in clinical trials

 ○ The need to accept VTE as a potential treatment-related SAE in clinical trials

 ○ The need for validated scoring systems that relate grade to outcome in clinical trials and clinical practice

 ○ The need for CRF design to be able to capture the ongoing nature of a VTE SAE in clinical trials.

 ○ The need for research to understand the significance of splanchnic DVT in cancer, especially in APC.

## **Box 2** Invisible VTE challenges

• Limitations because of the thrombosis

 ○ VTE can be ‘silent’

 ○ Natural history of silent VTE unknown

  ▪ Undiagnosed does not mean insignificant

• Limitations because of the cancer

 ○ Underlying cancer can obscure symptoms of VTE

  ▪ Undiagnosed does not mean insignificant

 ○ Type and stage of underlying cancer may dissuade clinician from extensive diagnostic work-up

• Limitations because of clinical processes and clinical trial method

 ○ Incidental VTE

  ▪ What is the contribution to mortality and morbidity of incidental VTE?

 ○ When to investigate atypical symptoms, for example, fatigue, for PE?

 ○ Natural history of silent VTE in cancer is unknown

  ▪ Does it need treatment?

  ▪ Grading and risk assessment unknown

## **Box 3** Recommendations

• Recommendations for clinical processes

 ○ A willingness to take patient views on risk benefit acceptance into account in decision making

 ○ A higher level of clinical suspicion

 ○ A greater understanding of the complexities of cancer-related VTE

 ○ Strategies for prevention, recognition and management of VTE should be developed

• Recommendations for research

 ○ A clinically relevant graded system for recording VTE in cancer trials

 ○ Systematic study of the clinical impact of co-incidental/silent VTE

 ○ Recording and reporting VTE with the same thoroughness as SAE

 ○ Continued research into optimal anticoagulation

 ○ Develop predictive biological assays of VTE

 ○ Unexpected early deaths in trial-eligible patients should be subject to post mortem
